# Learning a stable approximation of an existing but unknown inverse mapping: application to the half-time circular Radon transform

**DOI:** 10.1088/1361-6420/ad4f0a

**Published:** 2024-06-25

**Authors:** Refik Mert Cam, Umberto Villa, Mark A Anastasio

**Affiliations:** 1 Department of Electrical and Computer Engineering, University of Illinois Urbana–Champaign, Urbana, IL 61801, United States of America; 2 Oden Institute for Computational Engineering & Sciences, The University of Texas at Austin, Austin, TX 78712, United States of America; 3 Department of Bioengineering, University of Illinois Urbana–Champaign, Urbana, IL 61801, United States of America

**Keywords:** image reconstruction, deep learning, circular Radon transform

## Abstract

Supervised deep learning-based methods have inspired a new wave of image reconstruction methods that implicitly learn effective regularization strategies from a set of training data. While they hold potential for improving image quality, they have also raised concerns regarding their robustness. Instabilities can manifest when learned methods are applied to find approximate solutions to ill-posed image reconstruction problems for which a unique and stable inverse mapping does not exist, which is a typical use case. In this study, we investigate the performance of supervised deep learning-based image reconstruction in an alternate use case in which a stable inverse mapping is known to exist but is not yet analytically available in closed form. For such problems, a deep learning-based method can learn a stable approximation of the unknown inverse mapping that generalizes well to data that differ significantly from the training set. The learned approximation of the inverse mapping eliminates the need to employ an implicit (optimization-based) reconstruction method and can potentially yield insights into the unknown analytic inverse formula. The specific problem addressed is image reconstruction from a particular case of radially truncated circular Radon transform (CRT) data, referred to as ‘half-time’ measurement data. For the half-time image reconstruction problem, we develop and investigate a learned filtered backprojection method that employs a convolutional neural network to approximate the unknown filtering operation. We demonstrate that this method behaves stably and readily generalizes to data that differ significantly from training data. The developed method may find application to wave-based imaging modalities that include photoacoustic computed tomography.

## 1. Introduction

Image reconstruction involves generating an estimate of an object function from indirect measurements of the object, and the associated inverse problem is often ill-posed. Traditional image reconstruction methods can be classified as being direct or implicit [1]. Direct methods are based upon analytical solutions of the inverse problems that are established in a continuous setting where noise and discrete sampling effects are typically not explicitly addressed. In contrast, implicit methods produce solutions, or approximate solutions, of an inverse problem via the solution of a specified optimization problem. Such methods provide greater flexibility to account for physical factors such as measurement noise, and also provide a general framework for regularization. Relatively recently, a third category of image reconstruction techniques based on deep learning (DL) has emerged and gained considerable attention [2–11]. DL-based image reconstruction methods seek to regularize the solutions of inverse problems in a data-driven way, which could potentially improve upon the effectiveness of hand-crafted object priors that are incorporated in implicit reconstruction methods [12]. In the remainder of this article, DL-based reconstruction methods will refer to ones based on supervised learning [3, 13, 14].

However, it has been established that DL-based image reconstruction methods can suffer from hallucinations, instabilities, and poor generalization [15–17] when applied to find approximate solutions to ill-posed inverse problems for which a unique solution does not exist and/or cannot be stably computed. Within this context, hallucinations refer to false structures in the reconstructed image or the omission of true structures [18–21]. Hallucinations pose a significant concern for DL-based methods because they can sometimes look natural and be difficult to detect [21]. Moreover, when applied to data outside the training distribution, DL-based methods may produce erroneous results [21]. The robustness of DL-based methods has been studied and revealed the challenge of balancing the accuracy-stability trade-off; it has been shown that the occurrence of hallucinations and instabilities are non-rare events often encouraged by standard training procedures [15, 22, 23].

A fundamentally different and seemingly underexplored use case for DL-based image reconstruction is to learn a stable inverse mapping that is known to exist but is not yet analytically available in closed form [24–27]. Such problems can be solved by use of implicit reconstruction methods that generally involve the use of iterative solvers [28–30]. As an alternative, DL provides an opportunity to establish, in a data-driven way, a stable approximation of the inverse mapping. Because the sought-after mapping is known to exist and be stable, a DL-based method that can effectively learn an approximation of the inverse mapping is expected to generalize well to data that differ significantly from the training data. As such, the limitations of standard applications of DL that seek to find solutions of ill-posed inverse problems, as described above, could be circumvented. Additionally, such a learned approximation of the inverse mapping would eliminate the need to employ an implicit reconstruction method that can be computationally burdensome.

In this work, a DL-based image reconstruction method is systematically investigated for this use case. Specifically, the problem of image reconstruction from radially-truncated circular Radon transform (CRT) data, known as ‘half-time’ measurement data [29, 31, 32], is considered. The CRT arises in the physical modeling of modern wave-based medical imaging modalities such as photoacoustic computed tomography [33–36] (PACT) and ultrasound reflectivity tomography [37, 38]. It is known that half-time measurement data are sufficient for stable inversion of the CRT; however, the associated inverse mapping remains unknown in closed form. To address this, a quasi-analytic image reconstruction method of filtered backprojection (FBP) form is established, where a linear U-Net [39] is employed to approximate the unknown data filtering operation. The performance of the proposed learned reconstruction method for use with half-time data is compared to that of a direct inversion method [40] that employs untruncated measurement data. Moreover, systematic numerical studies are conducted to assess the stability and generalization performance of the proposed learned method.

While there is a large body of literature proposing DL-based FBP methods to regularize ill-posed inverse problems [6, 11, 41–43], only a very limited number of studies addresses the case of a well-posed inverse problem [11, 43, 44], which is the main novelty of the present work. When the inverse problem is ill-posed, the DL-based FBP methods aim to regularize the inverse problem and mitigate artifacts by learning an implicit prior from the training dataset. However, due to the absence of a unique stable inverse mapping for such problems, the generalizability of DL-based methods to out-of-distribution data is not assured [15, 22, 23]. As for the cases where the inverse problem is well-posed, a seminal work by Floyd in 1991 [44] has shown that an artificial neural network is able to learn a shift-invariant filtering operator whose response significantly resembles the ramp filter typically employed in FBP algorithm for classical Radon transform. A few subsequent studies have shown the potential of directly mapping the FBP algorithm for classical Radon transform onto deep neural network architectures [11, 43]. However, these studies were limited to the case where the analytical FBP formula is known. In contrast, this study addresses a well-posed inverse problem for which the FBP formula in closed form has *not* been reported. The novelty of this work is to approximate the unknown FBP formula using a DL-based method. This method is expected—and demonstrated in the numerical results—to be readily generalizable to data that differ from the training data as the sought-after mapping is known to exist and be stable. Consequently, it should be distinguished from previous research endeavors.

The remainder of the article is organized as follows. Section 2 provides a brief review of some mathematical properties of the CRT that are salient to the half-time CRT inversion problem. The discretized CRT is formulated as a mapping between finite-dimensional vector spaces in section 3 and the ability to stably invert the discretized CRT from half-time data is motivated. The proposed learned half-time FBP (HT-FBP) method is described in section 4, and the numerical studies and corresponding results are provided in sections 5 and 6, respectively. Finally, the paper concludes with a discussion in section 7.

## 2. Background

The CRT integrates a two-dimensional (2D) object over circular arcs centered at the boundary of a measurement disc that encloses the object [31, 36, 45]. The CRT inversion is relevant to reconstructing the initial wave equation data from its observed solutions at specific locations. Under the assumption of a homogeneous medium, the solution of a 2D wave equation can be expressed through CRT [36, 46]. In this section, the canonical CRT is introduced, the existing literature demonstrating the well-posedness of the half-time CRT reconstruction problem is summarized, and some image reconstruction techniques are reviewed.

### 2.1. Canonical CRT and unique recovery from half-time CRT data

Let $f(r, \theta) \in C_c(\mathbb{R}^2)$ denote a continuous 2D object function with a compact support inside a circle of radius *R*.

**Figure 1.**
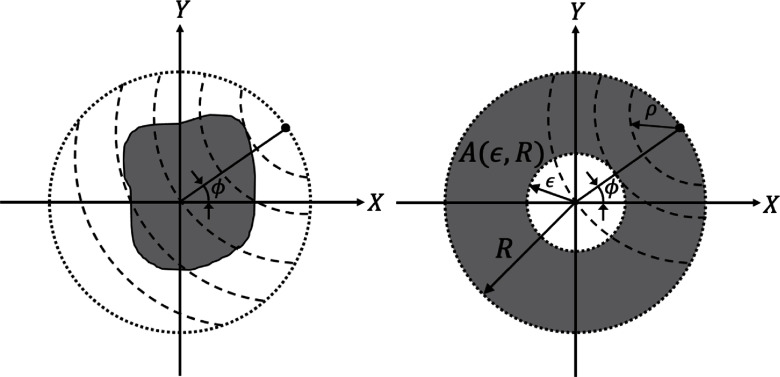
Schematics showing the paths of integration for a fixed view angle $\phi$ for the CRT (left panel) and half-time CRT (right panel). The shaded region in the right panel depicts the annular region $A(\epsilon,R)$ defined in theorem 1.

Definition 1. In its continuous form, the canonical CRT of $f(r, \theta)$ is defined as:

\begin{equation*} g(\rho, \phi) = \int_{\sigma(\rho, \phi)} f(r, \theta){\mathrm{d}}s,\quad \rho \in [0, 2R]\;\; {\mathrm{and}} \;\; \phi \in [0, 2\pi),\end{equation*}

where $g(\rho, \phi)$ is the line integral of $f(r, \theta)$ over a circular arc $\sigma(\rho, \phi)$, of radius $\rho$, and whose center lies on the boundary of the enclosing circle at an angle $\phi$. These variables are depicted in figure 1(left panel).

In certain imaging applications, the coordinate $\rho$ is ascribed a temporal interpretation [29]. As such, in this work, the CRT described above will be referred to as the *full-time* CRT, indicating that there is no truncation of the data function $g(\rho, \phi)$ with respect to $\rho$.

Definition 2. The *half-time* CRT of $f(r, \theta)$, denoted as $g_{\mathrm{ht}}(\rho, \phi)$ and depicted in figure 1(right panel), is defined as follows:

\begin{equation*} g_{\mathrm{ht}}(\rho, \phi) = \int_{\sigma(\rho, \phi)} f(r, \theta){\mathrm{d}}s,\quad \rho \in [0, R)\;\; \mathrm{and} \;\; \phi \in [0, 2\pi),\end{equation*}

where $g_{\mathrm{ht}}(\rho, \phi)$ is the line integral of $f(r, \theta)$ over a circular arc $\sigma(\rho, \phi)$ with radius $\rho$ and whose center lies on the boundary of the enclosing circle at an angle $\phi$, subject to the constraint that the radius $\rho$ is limited to $[0, R)$.

As such, the half-time CRT represents a radially truncated CRT in which integrations over half of the arcs through the object are acquired at each view angle $\phi$.

Range conditions for the CRT, including natural support, moment, and orthogonality, are specified in [47, 48]. Although the orthogonality constraints are considered analogous to the even symmetry property of the classical Radon transform, the range conditions of the CRT lack an explicit symmetry property. An implicit data symmetry has been demonstrated using a heuristic referred to as the ‘potato-peeler’ procedure in [30]. Conditions that ensure the unique recovery of an object function from CRT data are summarized in the following theorem [45]:

Theorem 1. Let $f(r, \theta) \in C_c(\mathbb{R}^2)$ be an unknown smooth function supported inside the annulus $A(\epsilon, R) = \{(r, \theta) : r \in (\epsilon, R), \theta \in [0, 2\pi)\}$, where $0 < \epsilon < R$. If $g(\rho, \phi)$ is known for $\phi \in [0, 2\pi)$ and $\rho \in [0, R-\epsilon]$, then $f(r, \theta)$ can be uniquely recovered in $A(\epsilon, R)$.

This theorem establishes the unique recovery of an unknown smooth function $f(r, \theta)$ that is compactly supported within the annulus $A(\epsilon, R)$ depicted in figure 1(right panel), when certain truncated CRT data are available.

### 2.2. Microlocal correspondence for the CRT

The stable recovery of singularities in the object function can be studied by microlocal analysis [49]. Discontinuities of a function are reflected in the decay properties of its Fourier transform [50]. The Fourier transform of a smooth function decays rapidly, while functions with discontinuities exhibit slower decay. The wavefront set characterizes the singularities of a function and is defined through localized analysis of its Fourier transform. By considering a compactly supported and infinitely differentiable window function $W(r, \theta)$ that goes to zero outside of some neighborhood of $(r_s, \theta_s)$, the decay properties of the Fourier transform can be studied in a localized region. The function $f_W(r, \theta) = W(r, \theta)f(r,\theta)$ shares the singularities of $f(r,\theta)$ near $(r_s, \theta_s)$. The direction of a singularity at $(r_s, \theta_s)$ in $f(r, \theta)$ is denoted by $\xi(r_s, \theta_s)$, which specifies the direction in which the Fourier transform does not decay rapidly. The wavefront set $WF(f)$ captures all such elements ${((r_s, \theta_s), \xi(r_s, \theta_s))}$ and fully characterizes the singularities in $f(r, \theta)$.

Microlocal correspondences establish the relationship between the wavefront set of the object function and the wavefront set of the corresponding tomographic data function. These correspondences provide necessary conditions for ensuring stable reconstruction in inverse problems. The microlocal correspondence for the CRT implies the following [24, 31, 51]:

Theorem 2. A wavefront set component $\{((r_s, \theta_s), \xi(r_s, \theta_s))\}$ of $f(r, \theta)$ is stably recoverable (or detectable) from CRT data if and only if there is an integrating circular arc passing through $(r_s, \theta_s)$ and its tangent is perpendicular to $\xi(r_s, \theta_s)$.

This theorem ensures the stable recovery of singularities of an object function from CRT measurements, given that the singularity intersects tangentially with an integrating circular arc. Consequently, it is clear that any singularity within the object function that resides inside the measurement disc can be stably reconstructed by use of half-time CRT data [29].

### 2.3. Image reconstruction techniques

Several image reconstruction formulae for the full-time CRT have been proposed [52], including ones based on series expansions [37] and FBP type methods [40, 53]. A generic FBP type inversion formula can be expressed as:

\begin{equation*} \tilde{f} = \mathcal{H^\dagger}\mathcal{F}g,\end{equation*}

where $\tilde{f}$, $\mathcal{H}^\dagger$, $\mathcal{F}$ correspond to the reconstructed object estimate, the adjoint of the CRT imaging operator, and the data space filtering operator, respectively. A review of reconstruction strategies for full-time CRT and its 3D generalization, the spherical Radon transform (SRT), is available in [54].

Direct reconstruction methods of FBP-type for the half-time CRT have not been reported. An expectation-minimization (EM) iterative algorithm was introduced to estimate a discrete object representation from half-time CRT data in [29]. In [45], an analytical solution for the half-time CRT inversion is presented; however, the method requires iterative solution of a Volterra equation. The existence, uniqueness, and stability of the solution as well as an ordered-subset EM-based algorithm to obtain a discrete object estimate have been presented in [28]. In [24], reconstruction formulae along with the uniqueness proof for radially truncated SRT inversion have been stated; however, implementation of these formulae require the iterative solution of a Volterra equation.

## 3. Discretized CRT

The sampling conditions for accurately recovering a continuous object function from a finite number of samples of CRT data are derived in [36]. Let the object function $f(r, \theta) \in C_c(\mathbb{R}^2)$ be essentially $b_0$-bandlimited. Here, the term ‘essentially $b_0$-bandlimited’ means that the Fourier transform of the object function is sufficiently confined to the disc of radius $b_0$ [36]. Then, under an equispaced sampling scheme, the required numbers of radial ($N_\rho$) and angular ($N_\phi$) samples to uniquely determine the object function with a small error are [36]:

\begin{equation*} N_\phi \unicode{x2A7E} 2Rb_0,\end{equation*}

\begin{equation*} N_\rho \unicode{x2A7E} 2Rb_0/\pi,\end{equation*}

which also correspond to the sampling conditions for the classic Radon transform [36].

Because $f(r, \theta)$ is essentially $b_0$-bandlimited, it can be accurately represented with a finite number of expansion functions [55, 56]. A variety of discretization methods [24, 36, 54, 57] can be applied to approximate the CRT forward problem in discrete form as

\begin{equation*} \boldsymbol{g} = \boldsymbol{H} \boldsymbol{f},\end{equation*}

where $\boldsymbol{g} \in \mathbb{R}^M$ denotes the sampled measured data, $\boldsymbol{f} \in \mathbb{R}^N$ is the finite-dimensional approximation of the object function, and $\boldsymbol{H} \in \mathbb{R}^{M \times N}$ represents the discretized CRT imaging operator. The discrete approximation of the half-time CRT inversion problem utilizes half of the radial samples of the CRT operator. Consequently, the same relationship remains applicable but the notation is updated to denote that only half of the radial samples are assumed recorded: $\boldsymbol{g_{ht}} = \boldsymbol{H_{ht}} \boldsymbol{f}$, where $\boldsymbol{H_{ht}} \in \mathbb{R}^{\frac{M}{2} \times N}$ is the half-time CRT operator and $\boldsymbol{g_{ht}} \in \mathbb{R}^{\frac{M}{2}}$ is the half-time data vector.

In the finite-dimensional setting, the existence of a unique $\boldsymbol{f}$ that corresponds to a half-time measurement vector requires the following conditions. First, the matrix, $\boldsymbol{H_{ht}}$, needs to be of full column-rank, implying that $rank(\boldsymbol{H_{ht}}) = N$, which ensures the absence of a nontrivial null space. In this case, the singular value decomposition (SVD) of $\boldsymbol{H_{ht}}$ can be represented as $\boldsymbol{H_{ht}} = \boldsymbol{U_{ht}}\boldsymbol{\Sigma_{ht}}\boldsymbol{V_{ht}}^T$, where $\boldsymbol{U_{ht}} \in \mathbb{R}^{\frac{M}{2} \times N}$ denotes a matrix with orthonormal columns, $\boldsymbol{\Sigma_{ht}} \in \mathbb{R}^{N \times N}$ is a diagonal matrix with positive entries $\sigma_i > 0$ for $i = 1,{\ldots},N$, and $\boldsymbol{V_{ht}} \in \mathbb{R}^{N \times N}$ is an orthonormal matrix. Second, the measurement vector should reside within the range space of $\boldsymbol{H_{ht}}$, resulting in a consistent system of equations.

**Figure 2.**
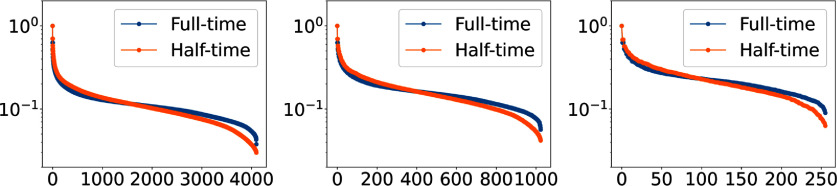
Normalized singular values of the discretized untruncated and half-time CRT operators for three measurement settings from table 1: **(First)** Setting I, **(Second)** Setting II, and **(Third)** Setting III. The largest singular value is scaled to 1 for both untruncated and half-time CRT operators. The spectra of $\boldsymbol{H_{ht}}$ and $\boldsymbol{H}$ demonstrate noteworthy similarity across each setting, providing empirical evidence towards the invertibility of the half-time CRT operator.

To empirically motivate the invertibility of the discrete half-time CRT operator $\boldsymbol{H_{ht}}$, three small scale operators were considered. To establish $\boldsymbol{H_{ht}}$, pixel expansion functions were employed and the continuous CRT operator was discretized by use of a variant of Siddon’s ray tracing algorithm [57, 58]. The full-time operator $\boldsymbol{H}$ was formed similarly. The $\ell^2$-norm condition numbers of $\boldsymbol{H_{ht}}$ and $\boldsymbol{H}$ for three different measurement configurations were computed, where in each case the number of measurements exceeded the dimension of the discretized object. The results, presented in table 1, reveal that, for all cases, the two operators are both full-rank and possess comparable $\ell^2$-norm condition numbers. Singular value decompositions of $\boldsymbol{H_{ht}}$ and $\boldsymbol{H}$ were also computed. Figure 2 shows the singular value plots, where the largest singular value is normalized to 1. For each measurement configuration, the singular value spectra of $\boldsymbol{H_{ht}}$ and $\boldsymbol{H}$ were found to be qualitatively similar.

**Table 1.** The $\ell^2$-norm condition numbers of discretized small-scale untruncated and half-time CRT operators for three different measurement settings.

Measurement setting	Grid size	Number of tomographic views	Number of arcs per view (untruncated)	Condition number of the untruncated CRT operator	Condition number of the half-time CRT operator
I	$64\times64$	128	200	2.881/0.109 = 26.49	1.789/0.054 = 33.41
II	$32\times32$	64	100	2.884/0.162 = 17.75	1.807/0.075 = 23.94
III	$16\times16$	32	50	2.885/0.259 = 11.14	1.841/0.116 = 15.89

## 4. Learned FBP method for use with half-time CRT data

The results reviewed in section 2 established that the half-time CRT in equation (2) can be stably inverted, while section 3 provided empirical evidence of the same for its suitably discretized version. In practice, a direct reconstruction method of FBP type is available for estimating the object function from discrete full-time measurements [53]; however, such a reconstruction method is not currently available for the half-time problem. In this section, a discrete FBP method is established for this purpose, in which the currently unknown data filtering operation is approximated by use of a learning-based procedure.

A discretized FBP technique for use with half-time CRT data can be formulated as $\boldsymbol{\tilde{f}} = \boldsymbol{H_{ht}}^T\boldsymbol{F}\boldsymbol{g_{ht}}$. In this equation, $\boldsymbol{\tilde{f}}$, $\boldsymbol{H_{ht}}^T$, ***F***, and $\boldsymbol{g_{ht}}$ represent the discrete recovery, backprojection operator, filtering operator, and the half-time CRT data, respectively. The optimal data filtering operator, denoted as $\boldsymbol{F_{opt}} \in \mathbb{R}^{\frac{M}{2} \times \frac{M}{2}}$, should satisfy the condition $\boldsymbol{H_{ht}}^T\boldsymbol{F_{opt}}\boldsymbol{H_{ht}} = \boldsymbol{I}_N$, where ***I***_*N*_ denotes the identity matrix in $\mathbb{R}^N$. This optimal filtering operator can be derived by utilizing the SVD of $\boldsymbol{H_{ht}}$ as $\boldsymbol{F_{opt}} = \boldsymbol{U_{ht}}\boldsymbol{\Sigma_{ht}}^{-2}\boldsymbol{U_{ht}}^T$. However, computing the SVD for large-scale matrices is computationally challenging [59, 60]. Therefore, an alternative approach involves formulating the optimal filtering operator as the solution to the following optimization problem:

\begin{equation*} \hat{\boldsymbol{F}} = \mathop{\mathrm{argmin}}\limits_{\boldsymbol{F}} ||\boldsymbol{H_{ht}}^T\boldsymbol{F}\boldsymbol{H_{ht}} - \boldsymbol{I}_N||^2_F,\end{equation*}

where, $||\cdot||_F$ denotes the Frobenius norm. Solving this problem often relies on matrix factorizations, which can also be computationally expensive [61, 62]. To address this and make the optimization problem computationally feasible, the problem can be approximated as a stochastic minimization problem within the context of randomized trace estimators [63, 64]:

\begin{equation*} \hat{\boldsymbol{F}} = \mathop{\mathrm{argmin}}\limits_{\boldsymbol{F}} \mathbb{E}_{\boldsymbol{f}}\left[||\boldsymbol{H_{ht}}^T\boldsymbol{F}\boldsymbol{H_{ht}}\,\boldsymbol{f} - \boldsymbol{f}||_2^2\right],\end{equation*}

where, the expectation $\mathbb{E}_{\boldsymbol{f}}[\cdot]$ is taken over a suitable probability distribution. Then, the stochastic minimization problem can be transformed into a learning-based algorithm within the framework of empirical risk minimization. By leveraging a training set consisting of independent identically distributed (i.i.d.) samples, $\mathcal{T} = \{(\boldsymbol{g}_i, \,\boldsymbol{f}_i)\}_{i = 1}^K$, where $\boldsymbol{g}_i = \boldsymbol{H_{ht}} \,\boldsymbol{f}_i$, a parameterized filtering operator denoted by $\boldsymbol{F}_{\phi}$ can be learned. The resulting minimization problem can be expressed as:

\begin{equation*} \hat{\boldsymbol{F}_{\phi}} = \mathop{\mathrm{argmin}}\limits_{\boldsymbol{F}_{\phi}} \frac{1}{|\mathcal{T}|} \sum_{\left(\boldsymbol{g}, \,\boldsymbol{f}\right) \in \mathcal{T}} ||\boldsymbol{H_{ht}}^T\boldsymbol{F}_{\phi}\boldsymbol{g} - \boldsymbol{f}\,||_2^2.\end{equation*}

**Figure 3.**
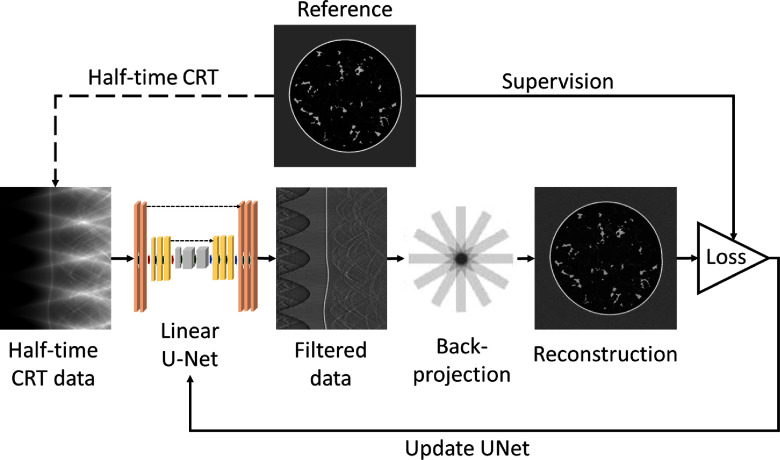
Illustration of the learned half-time FBP framework. The U-Net takes the half-time CRT data as input and outputs the filtered data that is later backprojected to form the reconstruction. MSE between the reconstruction and the reference was used as the loss function, and the U-Net was trained accordingly.

In this study, a linear 2D convolutional neural network (CNN) whose architecture is inspired by the U-Net, hereafter referred to as the linear U-Net, was trained to approximate the data filtering operation $\boldsymbol{F}_{\phi}$. The proposed approach employs the trained linear U-Net to map the half-time CRT data to its appropriately filtered version, which is subsequently backprojected into image space to form the object estimate, as depicted in figure 3. Nonlinearities commonly used in DL were intentionally omitted due to the linearity of the half-time CRT inversion problem.

The resulting half-time FBP method is appealing because it is readily interpretable and imaging operator aware due to the involvement of the backprojection operator. However, it is important to note that the proposed approach does not seek to exactly determine the unknown analytical filtering but only approximate it for at least two reasons. First, the proposed framework considers the discrete half-time CRT model instead of the underlying continuous model. In the discrete half-time CRT model, the continuous object is represented using expansion functions (e.g. pixels), which is merely an approximation of the continuous object function and not an exact representation. Second, the estimated filtering operator is limited by the representation capacity of the specific linear U-Net employed. Although a dense (i.e. fully connected) neural network layer could theoretically represent any linear discrete filtering operator, it becomes impractical for this problem due to the substantial memory requirements. For example, with 512 angles and 400 integrating arcs per angle, the dense layer would necessitate approximately $(512\times400)^2 \approx 4.2\times10^{10}$ parameters, consuming around 156 GB of memory in single-precision. As a result, the linear U-Net was chosen as a more feasible approach.

The employed linear U-Net architecture consists of two main components: the encoder and decoder [39]. The encoder progressively reduces the spatial dimension while increasing the number of channels, and the decoder performs the opposite operation. To preserve low-level features, the architecture includes skip connections that concatenate the encoder and decoder paths. To impose the linearity of the network, nonlinear components such as activation functions and max pooling operations were eschewed. In the linear U-Net implementation, a convolutional block in the encoder consists of two $5\times5$ convolutions followed by one $2\times2$ convolution with stride size equal to 2 for downsampling. The first convolutional block consists of 16 channels and after each downsampling step, the number of channels is doubled. The encoder consists of 6 convolutional blocks and is followed by the bottleneck. The bottleneck consists of two $5\times5$ convolutional layers with 1024 channels. The decoder follows the bottleneck, and each convolutional block in the decoder consists of a $2\times2$ upsampling operator followed by one $2\times2$ and two $5\times5$ convolutions. After each upsampling step, the number of channels is reduced in half, and the features from the same level of the encoder are concatenated with the features of the decoder. Finally, the output of the U-Net is generated by applying a $1\times1$ convolution with one output channel.

## 5. Numerical studies

Numerical studies were conducted to evaluate the reconstruction performance, generalization, and stability of the linear learned half-time FBP method. This section provides information about the data used and the details of the noiseless and noisy data studies. The impact of training data diversity on the learned approximate inverse mapping is also investigated in the numerical studies.

### 5.1. Dataset, preprocessing and training

The datasets utilized for this work include 1440 2D acoustic impedance images of breast tissue, extracted from three-dimensional stochastic numerical breast phantoms [65], 1440 2D computed tomography (CT) images from the Deep Lesion dataset [66], and 1440 2D piecewise constant slices extracted from an anatomically realistic mouse atlas [67]. Each image in the datasets is the size of $512\times512$ pixels. Normalization of the datasets was performed to scale the range of the images between 0 and 1. The datasets were split into training, validation, and test sets, with 1160, 140, and 140 images in each dataset, respectively. Hereafter, these sets will be referred to as the breast, CT, and mouse datasets for training, validation, and testing. In noiseless and noisy data studies, models were trained on the breast training set, validated with breast validation set and tested across the breast, CT, and mouse test sets. The breast test set served as the in-distribution test set, while the CT and mouse test sets were used to assess the models’ generalization to out-of-distribution data. For the study to investigate the impact of training data diversity, models were individually trained on the breast, CT, and mouse training sets, validated with the matching validation set, and tested on all test sets.

**Figure 4.**
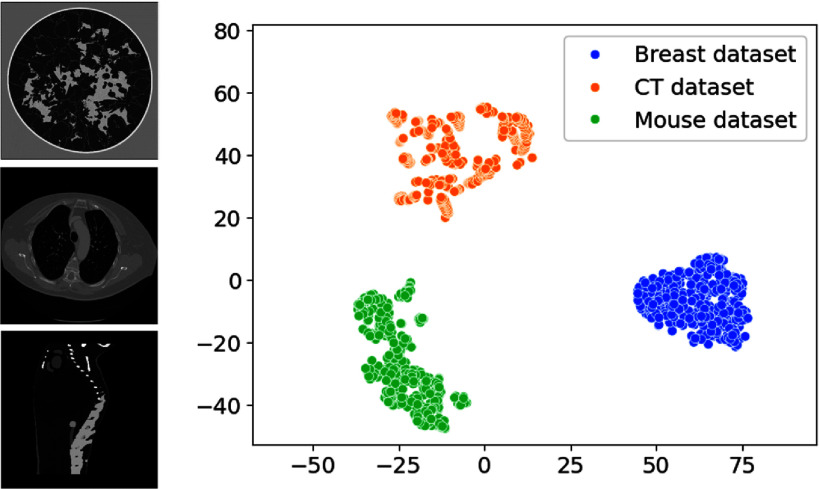
Left: examples of objects from the breast (top), CT (middle) and mouse (bottom) dataset. Gray-scale range is [0,1]. Right: t-SNE visualization of the datasets. Both the phantoms and the t-SNE projections underscore the dissimilarity between the datasets.

The left panel of figure 4 shows examples of phantoms from each dataset, while the right panel illustrates the associated 2D t-SNE visualization. The t-SNE is a technique for visualizing high-dimensional data by representing each data point as a point in a low-dimensional space, such as a 2D plot [68]. Both the visual inspection of the phantoms and the t-SNE visualization highlights the dissimilarity between the datasets. This is important because it will allow for meaningful assessments of the generalization performance of the linear learned HT-FBP method, which is expected to generalize well because a well-defined and stable inverse mapping is stipulated as described next.

A discrete half-time CRT with 512 tomographic views ($\phi$) and 400 circular integration arcs per view ($\rho$) was employed, which operates on a discrete grid of size $256\times256$. The number of tomographic views and integrations per view were chosen such that the imaging operator had a trivial null-space. The full-rankness of the half-time discretized CRT operator was verified through computing its largest and smallest singular values, which were found to be 1.777 and 0.026, respectively, resulting in an $\ell^2$-norm condition number of 67.80. In comparison, the smallest singular value of the corresponding full-time CRT operator with 800 circular integration arcs per view was 0.054, and its largest singular value was 2.881, resulting in an $\ell^2$-norm condition number of 53.62, comparable to that of the half-time CRT operator.

To enhance data variability during training, a data augmentation technique was applied, where the images were randomly rotated and either cropped or downsampled to $256\times256$ pixels at each epoch. The resulting images were then used to compute half-time CRT data with a size of $512\times400$. Since the CRT is periodic over the views with a period of 2 *$\pi$*; the first 128 views of the half-time CRT data, corresponding to $[0, \pi/2]$ angles, were patched to the end of the data; and the last 128 views, corresponding to $[3\pi/2, 2\pi]$ view angles, were patched to the beginning of the data. This pre-processing step ensured that the proximity between the tomographic views in the first and fourth quadrants was preserved.

The method was implemented in the TensorFlow 1.15.0 environment [69]. In order to facilitate backpropagation through the backprojection operator in the proposed method, it was encapsulated as a TensorFlow operation, and its adjoint was explicitly defined. During the training of the U-Net, the mean squared error (MSE) between the reconstruction and the reference image was used as the loss function. The weights of the U-Net were initialized randomly and trained using the ADAM optimizer [70]. A constant step size of 10^−5^ was used, and training continued until no further decrease in the validation loss was observed for 50 consecutive epochs.

### 5.2. Noiseless data study

A comparison was conducted between the linear learned HT-FBP method and a discretized analytical full-time FBP (FT-FBP) technique that operated on full-time data as a reference (benchmark) method. It is expected that the proposed learned HT-FBP can achieve image reconstructions of comparable quality to FT-FBP, when an appropriate network architecture and training procedure is implemented. Additionally, two alternative DL-based methods were considered to support the key features of the proposed method, namely the linearity of FBP and learning the mapping in data domain (as opposed to the image domain). Specifically, the alternative methods included a nonlinear half-time image-to-image U-Net, which implemented a learned post-processing mapping, and a nonlinear learned HT-FBP method. The image-to-image U-Net was primarily utilized to investigate the performance when a mapping is learned in the image domain (instead of the data domain); the nonlinear learned HT-FBP was selected to explore the scenario where linearity is not imposed. Network architecture parameters (number of levels, convolution channels, number of learnable parameters) similar to those used for the proposed learned HT-FBP method were chosen based on a manual architecture search aiming at balancing the trade-off between capacity, generalizability and parsimony.

**Analytical FT-FBP:** The analytical FT-FBP is a direct reconstruction method that maps full-time CRT data to corresponding images. In this study, it serves as the benchmark against which the linear learned HT-FBP method is evaluated. If the reconstructions produced by the linear learned HT-FBP method closely resemble those obtained with the analytical FT-FBP, it indicates that the former can effectively approximate the unknown half-time CRT inversion mapping. The discretized analytical FT-FBP formula was implemented based on the algorithm presented in [53].

**Half-time nonlinear image-to-image U-Net:** This alternative method was chosen to assess the degradation in generalizability when the mapping is learned in the image domain as opposed to the data domain (as in the proposed method). Specifically, the half-time nonlinear image-to-image U-Net takes the backprojection of the half-time CRT data as input and generates the final recovery as output. It has the same convolutional layers as the filtering U-Net of the linear learned HT-FBP but it employs rectifier linear unit (ReLU) activation functions after the convolutional layers. Configurations with a number of convolutional filters equal to 1/8, 1/4, 1/2, 1, 2, and 4 times the number of filters used in linear learned HT-FBP were assessed. It was observed that configurations with fewer filters (1/8, 1/4, 1/2) resulted in underfitting for both the in-distribution and out-of-distribution datasets. Configurations with more filters (2, 4) resulted in overfitting for the out-of-distribution datasets. Consequently, the study presents results from the configuration with the same number of filters as the linear learned HT-FBP’s method, which represents the best trade-off between capacity (i.e. accuracy on the training set), generalizability, and parsimony in the number of trainable weights.

**Nonlinear learned HT-FBP:** This alternative method was implemented to evaluate the importance of preserving the linearity of the half-time CRT inversion map in the learned HT-FBP method. In the nonlinear learned HT-FBP method, the learned data filtering operation is allowed to be nonlinear by employing a U-Net with leaky ReLU (alpha = 0.3) activation functions after each convolutional layer. The model has the same number of trainable parameters as the proposed linear learned HT-FBP method and it was trained in a similar manner.

### 5.3. Noisy data study

This study investigates the performance of the linear learned HT-FBP method in comparison to the analytical FT-FBP method in the presence of data inconsistency. To introduce data inconsistency, i.i.d. Gaussian noise was added to the CRT data. Specifically, the standard deviation of the Gaussian noise was set to be 1% of the maximum value of the ensemble of CRT data for each test set.

When dealing with data inconsistency, it is customary to employ apodization strategies in conjunction with direct image reconstruction techniques. In the conducted experiments, Gaussian smoothing was utilized as the apodization method for both the linear learned HT-FBP and analytical FT-FBP methods. Gaussian smoothing was applied along the radial samples of the data for each tomographic view. Two different window sizes were employed for Gaussian smoothing: *$\sigma$* = 2 and *$\sigma$* = 4. To avoid boundary artifacts when convolving with the Gaussian window, the data were extended by reflection outside the interval $[0,R)$ for the half-time method.

### 5.4. Investigation of the impact of training data diversity on the learned approximate inverse mapping

Given the unique and stable nature of the sought-after inverse mapping, it is expected that the accuracy of the learned mapping approximation should remain largely unaffected by the choice of the training dataset. This is based on the assumption that, as long as the span of training data is the range of the imaging operator $\boldsymbol{H_{ht}}$, the linear learned HT-FBP method can capture the relationship between half-time sinogram data and the object representation. To test this hypothesis, the linear learned HT-FBP method was trained separately with breast, CT, and mouse training sets. The efficacy of the learned approximate mapping was then tested against all test sets individually, monitoring for any discrepancies in MSE and SSIM values. Concurrently, image-to-image U-Net was trained similarly with breast, CT, and mouse training sets; and the MSE and SSIM metrics were compared.

### 5.5. Evaluation strategy

The performance of the linear learned HT-FBP method was evaluated and compared to the other methods using MSE and structural similarity index (SSIM). Specifically, for assessing the generalization capability of the proposed method, the MSE and SSIM metrics computed over out-of-distribution test sets were examined and compared with the results achieved by other techniques. In addition to this quantitative analysis, reconstructed images were visually inspected to identify any potential instabilities and/or hallucinations.

## 6. Results

### 6.1. Noiseless data study

**Figure 5.**
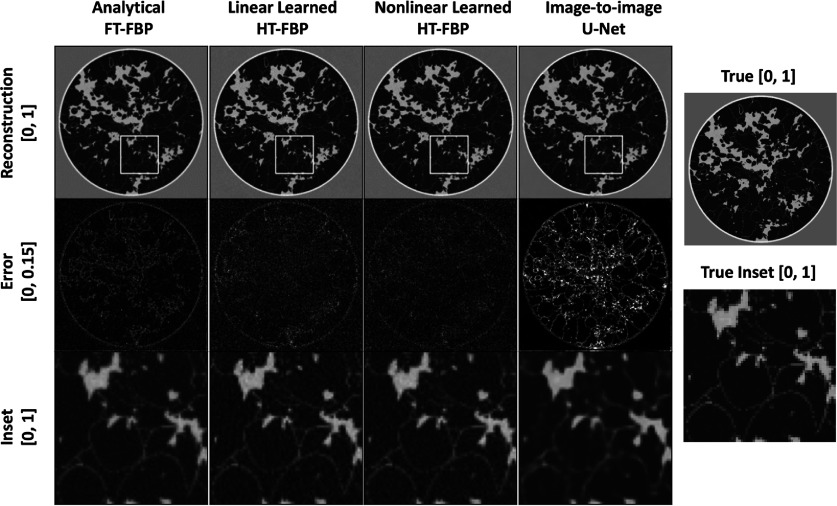
Sample reconstructions from in-distribution test set with analytical FT-FBP, proposed linear learned HT-FBP, nonlinear learned HT-FBP, half-time nonlinear image-to-image U-Net (first row); their absolute error images (second row); and insets of the reconstructed images within the marked regions (third row). The gray scale range is $[0,1]$ for the reconstructed images and the inset; $[0, 0.15]$ for the error images. For the in-distribution test set, the analytical FT-FBP, linear learned HT-FBP, and nonlinear learned HT-FBP techniques yield visually similar results. Although the nonlinear half-time image-to-image U-Net does not recover as effectively as the other methods, it still produces a reconstruction that is consistent with the true image.

**Figure 6.**
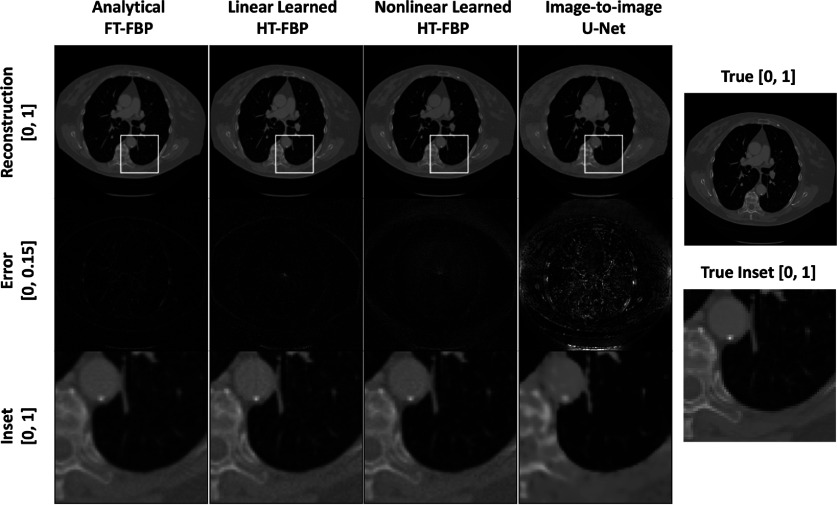
Sample reconstructions from out-of-distribution test set I with analytical FT-FBP, proposed linear learned HT-FBP, nonlinear learned HT-FBP, half-time nonlinear image-to-image U-Net (first row); their absolute error images (second row); and insets of the reconstructed images within the marked regions (third row). The gray scale range is $[0,1]$ for the reconstructed images and the inset; $[0, 0.15]$ for the error images. The analytical FT-FBP and the linear learned HT-BP techniques generate reconstructions with high similarity for this out-of-distribution test set. Although nonlinear learned HT-FBP recovery looks close to the linear learned HT-FBP recovery; when the absolute error image is examined, it is seen that the method is biased to generate circularly shaped objects. On the other hand, the image-to-image U-Net approach suffers from more severe hallucinations, such as missing bone structures.

**Figure 7.**
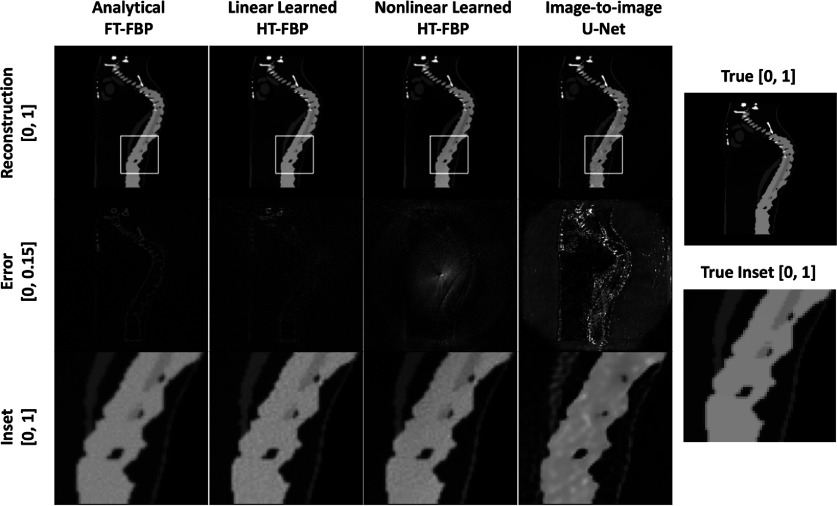
Sample reconstructions from out-of-distribution test set II with analytical FT-FBP, proposed linear learned HT-FBP, nonlinear learned HT-FBP, half-time nonlinear image-to-image U-Net (first row); their absolute error images (second row); and insets of the reconstructed images within the marked regions (third row). The gray scale range is $[0,1]$ for the reconstructed images and the inset; $[0, 0.15]$ for the error images. Similar to the results obtained from the out-of-distribution test set I, both analytical FT-FBP and linear learned HT-FBP exhibit recoveries that closely resemble the true image. However, the nonlinear learned HT-FBP and image-to-image U-Net methods demonstrate limitations as they tend to introduce hallucinations in the reconstructed images.

Examples of images reconstructed by use of the different methods from both the in-distribution (breast test set) and out-of-distribution test sets (CT and mouse test sets) are presented in figures 5–7. Hereafter, for the noiseless and noisy data studies, the CT test set and mouse test set are referred as out-of-distribution test set I and out-of-distribution test set II, respectively. For the in-distribution test set, all techniques yielded visually similar results. However, when applied to the out-of-distribution test sets, the half-time image-to-image U-Net and the nonlinear learned HT-FBP exhibited instabilities and/or hallucinations. The nonlinear learned HT-FBP tended to recover circularly shaped objects, possibly due to the training set consisting of circularly-shaped breast impedance phantoms. On the other hand, the image-to-image U-Net demonstrated more severe hallucinations, such as missing bones and introducing wavy textures in the reconstructions. This is possibly because it lacks information about the imaging operator and heavily relies on learned features in the image domain, making it more susceptible to generating false structures. In contrast, the linear learned HT-FBP consistently produced accurate images for both the in-distribution and out-of-distribution test sets, similar to the analytical FT-FBP, indicating its robust generalization performance. Furthermore, the robust generalization performance of the linear learned HT-FBP relative to the nonlinear learned HT-FBP method demonstrates the importance of maintaining linearity in the proposed method.

**Figure 8.**
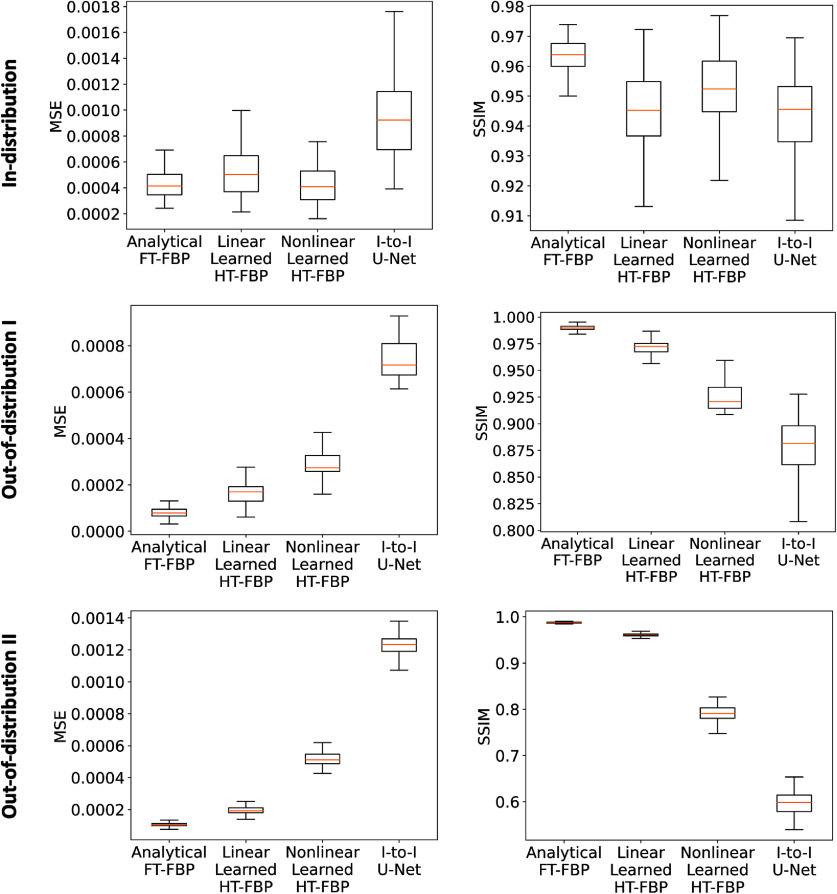
MSE (left) and SSIM (right) performance metrics of the analytical FT-FBP, linear learned HT-FBP, nonlinear learned HT-FBP, half-time image-to-image (I-to-I) U-Net, over in-distribution test set (first row), out-of-distribution test set I (second row), and out-of-distribution test set II (third row). The results demonstrate that all methods perform similarly to the analytical FT-FBP when evaluated on the in-distribution test set. However, for the out-of-distribution test sets, only the linear learned HT-FBP maintains comparable performance to the analytical FT-FBP. On the contrary, the nonlinear learned HT-FBP and the half-time image-to-image U-Net exhibit significantly poorer performance.

The MSE and SSIM values corresponding to the reconstructed images were computed for both in-distribution and out-of-distribution test sets. These results are illustrated in figure 8. Notably, the linear learned HT-FBP achieved close performance to the analytical FT-FBP across all test sets, extending beyond the specific image distribution it was trained on. This observation demonstrates that the linear learned HT-FBP possesses high generalizability performance, similar to the analytical method (FT-FBP). This corroborates that the proposed method effectively approximates the unknown half-time CRT data filtering operation. On the other hand, the nonlinear learned HT-FBP method and image-to-image U-Net performed similarly to the analytical FT-FBP for the in-distribution test set, but their performance significantly deteriorated when applied to out-of-distribution test sets.

### 6.2. Noisy data study

**Figure 9.**
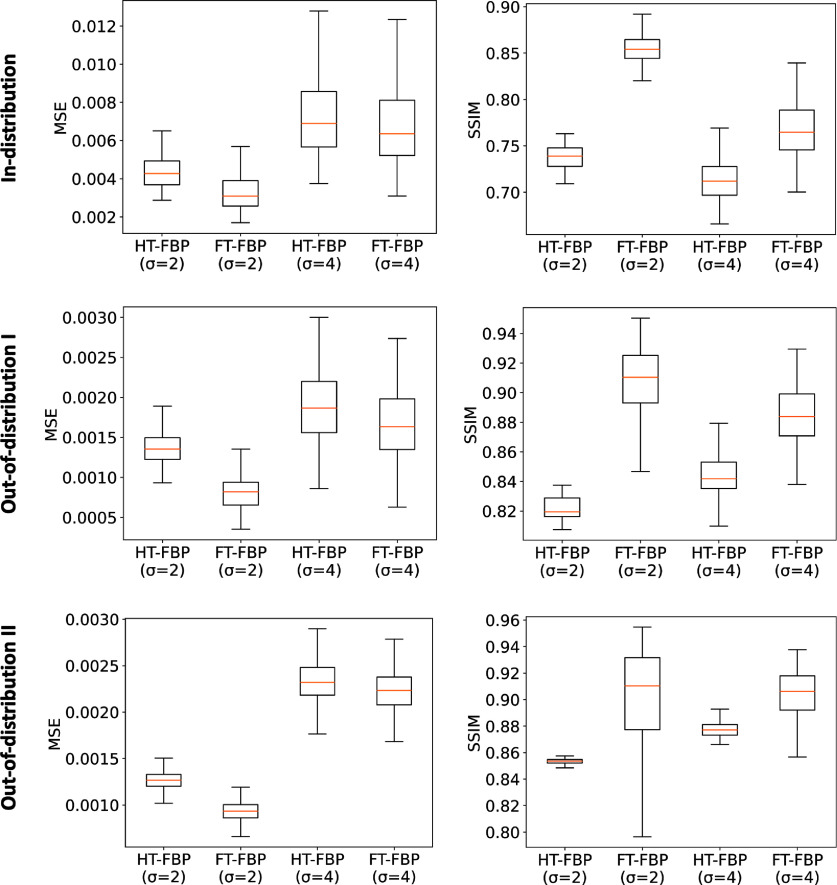
MSE (left) and SSIM (right) performance metrics of the apodized linear learned HT-FBP and apodized analytical FT-FBP, over in-distribution test set (first row), out-of-distribution test set I (second row), and out-of-distribution test set II (third row). The results indicate that the apodized linear learned HT-FBP achieves close performance to the apodized analytical FT-FBP across all test sets.

**Figure 10.**
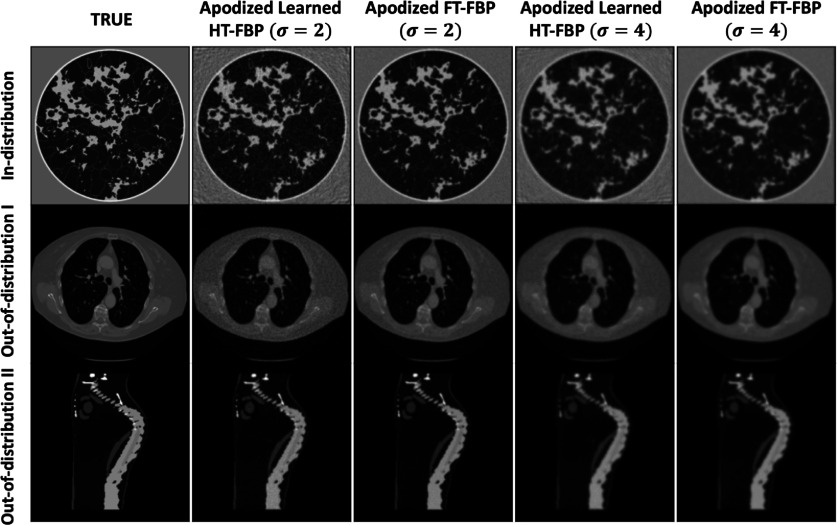
Sample reconstructions with apodized learned HT-FBP (linear) and analytical FT-FBP for noisy data from in-distribution test-set (first row), out-of-distribution test set I (second row), and out-of-distribution test set II (third row). The gray scale range is $[0,1]$ for the reconstructed images and the inset; $[0, 0.15]$ for the error images. The images are organized into columns, with the second and third columns displaying recoveries using a Gaussian window size, *$\sigma$*, set to 2, and the fourth and fifth columns show recoveries with *$\sigma$* = 4. Remarkably, regardless of the test set used, the apodized linear learned HT-FBP produces visually similar reconstructions compared to those generated by the apodized analytical FT-FBP.

The MSE and SSIM values corresponding to the images reconstructed from noisy data by use of the apodized linear learned HT-FBP and apodized analytical FT-FBP were computed. The results are presented in figure 9 for all the test sets. Although the apodized linear learned HT-FBP exhibited higher MSE and lower SSIM values compared to the apodized FT-FBP, in most cases the median value for the apodized linear learned HT-FBP fell within the range between the minimum and maximum values achieved by the apodized FT-FBP. This outcome was anticipated since the full-time data incorporates twice as many measurements as the half-time data. Sample reconstructions are shown in figure 10. For both apodization levels, *$\sigma$* = 2 and *$\sigma$* = 4, the apodized linear learned HT-FBP method yields visually similar reconstructions to those produced by the apodized analytical FT-FBP method across all test sets. This observation suggests that the proposed method not only exhibits robustness against data inconsistency but, importantly, demonstrates robust generalization performance even in the data inconsistent scenario. This behavior can be attributed to the fact that the considered inverse problem is well-posed and therefore the linear learned HT-FBP method is able to approximate the underlying unique and stable inverse mapping that is not dependent on the training data.

### 6.3. Investigation of the impact of training data diversity on learned approximation of inverse mapping

**Figure 11.**
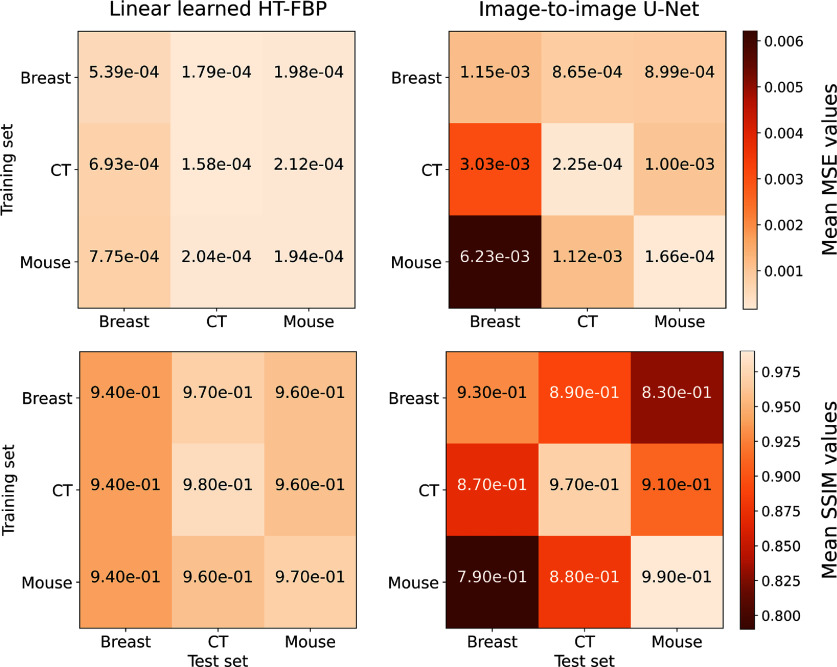
Comparative analysis of MSE and SSIM metrics for breast, CT, and mouse test sets, providing insight into the impact of training data diversity on the performance of the learned half-time FBP and image-to-image U-Net methodologies. The learned half-time FBP demonstrates robustness against the choice of the training set, whereas the image-to-image U-Net exhibits significant performance degradation when faced with test sets dissimilar to its training data.

Figure 11 presents the mean MSE and SSIM values for images reconstructed with the linear learned HT-FBP and image-to-image U-Net, trained on different datasets. The figure reveals that MSE and SSIM values for the linear learned HT-FBP remain relatively stable across varying training sets, which is expected. This stability indicates that the training set diversity does not substantially alter the accuracy of the linear learned HT-FBP. Nevertheless, a slight enhancement in MSE and SSIM is observable when the test set is sampled from the same distribution as the training set, suggesting that the linear learned HT-FBP method does not exactly learn the unique inverse mapping, but it does approximate the inverse mapping effectively through minimization over the training set samples. In stark contrast, the image-to-image U-Net demonstrates limited generalization, performing less optimally on test sets that are out-of-distribution. The image-to-image U-Net’s apparent shortfall in generalization might be associated with its training focus within the image domain, which predominantly concentrates on features in the image domain, leading to a reduced capacity for adapting to out-of-distribution datasets.

## 7. Conclusion

The sufficiency of half-time CRT data for unique and stable estimation of the object function has been established in previous works. However, an analytical formula of the FBP type remains unreported. This study explored the use of a linear learned FBP method for use with half-time CRT data. The findings indicate that the proposed method can accurately approximate the unknown data filtering operation in FBP and exhibits robust generalization performance, even when the test data significantly deviates from the training data. This holds true in both data consistent scenarios and when additive i.i.d. Gaussian noise is introduced.

It is important to note that most DL-based image reconstruction methods are developed to find approximate solutions to ill-posed inverse problems. This can lead to unstable learned methods that are prone to produce hallucinations. In contrast, this study addresses an inverse problem where the conditions for existence, uniqueness, and stability of the solution of the inverse problem are mathematically known to be satisfied, but a closed form inversion formula is yet to be reported. The study demonstrates that a stable, readily generalizable, and interpretable learning-based recovery technique can be developed for such problems. It also underscores how mathematical principles can guide the reliable application of DL in image reconstruction.

This study is anticipated to stimulate further research for cases where a unique inverse mapping is known to exist, but the analytical closed form formula remains unspecified. When the implicit methods are computationally demanding to employ, suboptimal direct methods are often utilized in practice for such inverse problems. For instance, hemispherical scanning geometry is frequently used for breast photoacoustic computed tomography, which is known to provide sufficient information to uniquely and stably estimate the object if the object is located in the convex-hull of the scanning geometry [28–30, 71]. However, an analytical closed form formula has only been reported for the spherical measurement geometry setting. Even though it is suboptimal for hemispherical scanning geometry, it is commonly used as the implicit methods are computationally burdensome. Therefore, a method similar to the one implemented in this study might be employed in future studies, potentially significantly improving the image quality for volumetric breast photoacoustic tomography imaging.

## Data Availability

The data that support the findings of this study are openly available at the following URL/DOI: https://figshare.com/articles/dataset/Results/24085827 [72].
